# Case 6 - Woman with Ischemic Heart Disease Admitted due to Chest Pain and Shock

**DOI:** 10.5935/abc.20180231

**Published:** 2018-12

**Authors:** Rafael Amorim Belo Nunes, Hilda Sara Montero Ramirez, Vera Demarchi Aiello

**Affiliations:** Instituto do Coração do Hospital das Clínicas da Faculdade de Medicina da Universidade de São Paulo (InCor-HC-FMUSP), São Paulo, SP - Brazil

**Keywords:** Myocardial Ischemia, Myocardial Infarction, Chest Pain, Cardiac Catheterization;Thromboembolism, Shock, Cardiogenic

A 67-year-old woman sought emergency medical care due to prolonged chest pain. In April 2009 the patient had prolonged chest pain and at that time she sought medical care. She was admitted at the hospital and diagnosed with myocardial infarction.

The patient had hypertension, diabetes mellitus, dyslipidemia and was a smoker.

During the patient's evolution, after the myocardial infarction, she was submitted to a coronary angiography in, which disclosed the presence of lesions with 70% obstruction in the right coronary, anterior descending and circumflex arteries. A left ventriculography revealed apical akinesia with signs of intracavitary thrombus in that region.

The echocardiogram (May 2009) disclosed ventricular dysfunction accentuated by diffuse hypokinesis, with a 28% left ventricular ejection fraction. Clinical and drug treatment was recommended to the patient.

The patient's evolution was asymptomatic until October 2009, when she had a cerebrovascular accident, with motor sequela.

On December 30, 2009, the patient had an episode of severe chest pain that lasted for one hour and she sought medical care.

At the physical examination, the heart rate (HR) was 100 beats per minute, blood pressure was 100/60 mmHg. Pulmonary assessment was normal. The heart examination disclosed a ++/ 6+ systolic murmur in the mitral area. The remainder of the physical examination was normal. The electrocardiogram (1h 19 min; Dec 30, 2009) showed sinus rhythm, HR of 103 bpm, PR interval of 122 ms, QRS duration of 159 ms, QT interval of 367 ms, and corrected QT of 480 ms.

There was left atrial overload, low voltage of the QRS complex in the frontal plane, probable inferior electrically inactive area, and left bundle branch block (Figure 1). Chest x-ray disclosed the presence of a large pleural effusion in the right hemithorax.

**Figure 1:**
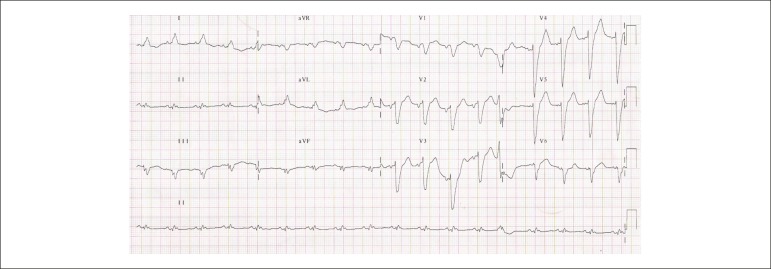
Electrocardiogram - Sinus rhythm, low voltage of the QRS complex in the frontal plane, electrically inactive area in the inferior wall and left bundle branch block.

The laboratory tests showed hemoglobin 13 g/dL, hematocrit 40%, MCV 91 fL, leukocytes 12,400/mm^3^ (66% neutrophils, 1% eosinophils, 1% basophils, 19% lymphocytes and 13% monocytes), 421,000/mm^3^, total cholesterol 228 mg/dL, HDL-cholesterol 35 mg / dL, LDL-cholesterol 162 mg/dL, triglycerides 157 mg/dL, CK-MB mass 5.63 ng / mL, Troponin I 0.21 ng/mL, urea 33 mg/dL, creatinine 0.66 mg/dL, sodium 137 mEq/L, and potassium 3.4 mEq/L. Venous blood gasometry showed pH 7.46, pCO_2_ 39.3 mmHg, pO_2_ 36.3 mmHg, O_2_ saturation 62.7%, bicarbonate 27.7 mEq/L and base excess 4.1 mEq/L.

Approximately two hours after hospital admission, she had seizures and cardiac arrest with pulseless electrical activity, reversed in 5 min.

The electrocardiogram after the cardiac arrest (4:18 am; Dec 30, 2009) showed a HR of 64 bpm, absence of P waves, and left bundle branch block. The QRS complex alteration, in relation to the previous tracing, was a positive QRS complex in the V6 lead (Figure 2).

**Figure 2:**
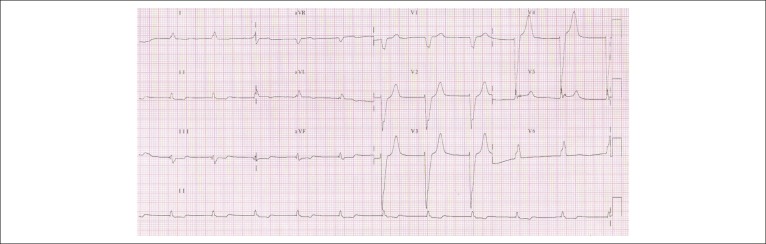
Electrocardiogram - Sinus rhythm, left bundle branch block and positive T waves on an also positive derivative of the QRS complex.

She had a new cardiac arrest 20 min later, which was also reversed. After half an hour, a new episode of cardiac arrest occurred, which was irreversible, and the patient died (5:45 am; Dec 30, 2009).

## Clinical aspects

This patient is a 67-year-old woman with cardiovascular risk factors and ischemic cardiomyopathy, with severe left ventricular systolic dysfunction. Cardiac catheterization disclosed multivessel coronary disease and apical akinesis with an intracavitary thrombus. During outpatient follow-up, clinical treatment was chosen, possibly influenced by the patient's clinical status, as well as the characteristics of the coronary anatomy.

The indication of surgical treatment with myocardial revascularization in patients with coronary heart disease with heart failure and severe left ventricular systolic dysfunction is still debatable, but recent data from the STICH study suggest a long-term survival benefit in patients undergoing myocardial revascularization.^1^

During follow-up in October 2009, the patient had a clinical picture suggestive of a cerebrovascular accident that may have been of atherothrombotic origin due to the multiple cardiovascular risk factors or of cardioembolic origin, associated with intracavitary thrombi.

In December 2009 the patient was admitted to the emergency room with acute chest pain. She had mild tachycardia and borderline systolic blood pressure of 100 mmHg. The electrocardiogram showed sinus tachycardia, left atrial overload and left bundle branch block.

In patients with acute chest pain and electrocardiogram with acute or undetermined left bundle branch block, the possibility of acute myocardial infarction should be considered, especially in case of hemodynamic instability. Criteria such as those proposed by Sgarbossa et al.,^2^ and Smith et al.,^3^ modified by other authors can contribute to the diagnostic accuracy improvement in this context.^2,3^ However, one should consider that the occurrence of left bundle branch block is more commonly a marker of previous structural heart disease.

The patient had a cardiorespiratory arrest with pulseless electrical activity (PEA) within a short time after hospital admission. In cases of acute myocardial infarction, PEA can occur in patients with severe ventricular dysfunction and cardiogenic shock and/or mechanical complications such as rupture of the left ventricular free wall with cardiac tamponade, papillary muscle rupture and / or severe dysfunction and acute interventricular septal defect.

Other conditions should be considered in patients with acute chest pain who present with rapid clinical deterioration such as aortic dissection and pulmonary thromboembolism. The chest x-ray showed a massive pleural effusion in the right hemithorax, although this finding was not readily apparent at the physical examination. In this patient, pleural effusion may be due to chronic heart failure decompensation but may also be associated with other conditions, such as rheumatologic diseases, tuberculosis or pleural carcinomatosis due to neoplasias. The last two conditions mentioned here are not uncommon in patients with chronic heart diseases.

Additionally, massive pleural effusions may coexist, in some conditions, with pericardial involvement and consequent cardiac tamponade.^4^ Pleural effusion may also be present in patients with acute aortopathies, such as dissection of the aorta and aortic ulcer with associated rupture, but usually the most frequent effusion is located in the left pleural space as a consequence of the aortic anatomy. (Dr. Hilda Sara Montero Ramirez)

**Main hypothesis:** Acute myocardial infarction complicated by cardiogenic shock. (**Dr. Hilda Sara Montero Ramirez**)

**Differential diagnoses:** Cardiac tamponade, Pulmonary thromboembolism and Dissection of the aorta. (**Dr. Hilda Sara Montero Ramirez**)

## Necropsy

**Figure 3:**
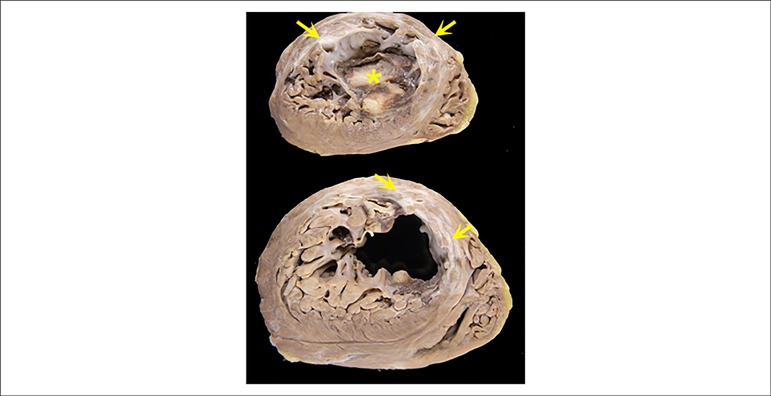
Cross-sections of the heart at the level of the ventricles (short axis) showing previous transmural infarctions in the anterior and septal walls (arrows). These same places show thinning of the wall and, localized slight dilatation (aneurysm). There is also a cavitary thrombus in the ventricular apex (asterisk).

The heart weighed 422 g and showed increased volume, with cross-sections (short axis of the ventricles) disclosing a healed transmural myocardial infarction in the left ventricular anterior and septal walls. There was wall thinning and fibrosis, with antero-apical aneurysm and thrombus at the apex (Figure 3). Signs of a previous systemic thromboembolism, with previous renal and cerebral infarctions were also found, with the latter being a cavitated infarction affecting the temporal and occipital regions of the left cerebral hemisphere.

The aorta and coronary arteries showed marked atherosclerotic involvement, with ulcerated plaques in the aorta and obstructions > 70% in the initial and middle thirds of the anterior interventricular branch of the left coronary artery and between 50 and 70% in the circumflex branch of the same artery and in the right coronary artery. Signs of congestive heart failure were found in the lungs and liver.

**Figure 4:**
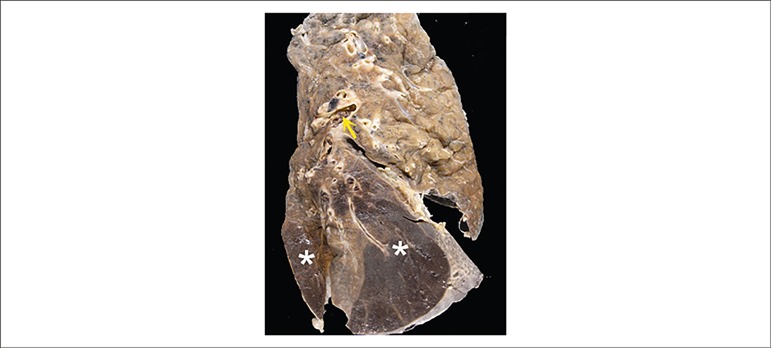
Right lung cross-section at its long axis showing the presence of thromboembolism in the central branch of the pulmonary artery (arrow). At the base, there are two triangular areas (asterisks) where the parenchyma is homogeneous and reddish in color, corresponding to recent pulmonary infarctions.

**Figure 5:**
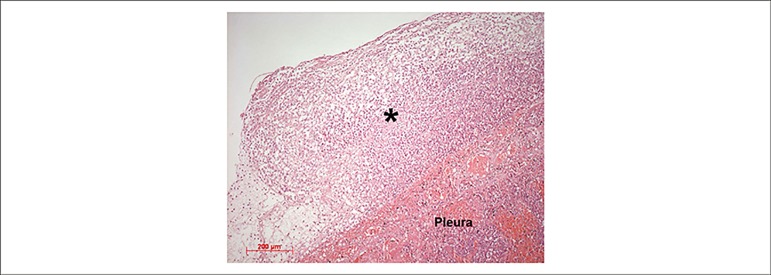
Photomicrography of the right pleura showing neutrophilic exudate on the surface (asterisk), characterizing acute pleuritis. Hematoxylin-eosin staining, objective magnification = 10X.

The terminal cause of death was pulmonary thromboembolism on the right, with infarction organization at the pulmonary base (Figure 4). The right pleura showed fibrin deposits and the histological analysis showed acute fibrinous pleuritis (Figure 5). There was also pleural effusion on the right (500mL of citrine-colored fluid) (**Prof. Dr. Vera D. Aiello**).

## Anatomopathological diagnoses

- Ischemic heart disease with healed transmural infarctions in the anterior wall and ventricular septum and anteroseptal aneurysm.
- Apical thrombus in the left ventricle.
- Systemic and coronary atherosclerosis of moderate to high degree.
- Previous infarctions in the kidneys and in the temporal and occipital cortex of the left cerebral hemisphere.
- Pulmonary thromboembolism on the right, with recent pulmonary infarction.
- Acute fibrinous pleuritis on the right, with pleural effusion (500mL) (**Prof. Dr. Vera D. Aiello**)

## Comments

The patient described herein sought emergency care with chest pain and was known to have ischemic heart disease. The clinical investigation for acute infarction was inconclusive and the patient died less than 24 hours after hospital admission.

Necropsy showed previous infarctions and signs of congestive heart failure. We found no evidence of a recent infarction and attributed the chest pain to the finding of a recent pulmonary thromboembolism on the right, with pulmonary infarction and acute fibrinous pleuritis.

In a study carried out at our institution, which assessed the agreement between clinical diagnoses and necropsy findings, the greatest discrepancy occurred in cases of pulmonary thromboembolism (34.1%).^5^ (**Prof. Dr. Vera Demarchi Aiello**)
